# Characterization of a *Salmonella* Phage-Derived Endolysin LysSPYS2 and Its Combined Activity with Polymyxin B and Magnolol

**DOI:** 10.3390/microorganisms14102407

**Published:** 2026-10-21

**Authors:** Yaxiong Song, Yaozhong Hu, Xiaoyi Wang, Yi Wang, Yousi Fu, Yali Wang, Wentao Gu, Yuanyifei Wang, Xinyang Li, Shuangqing Liu, Shuo Wang

**Affiliations:** 1 Tianjin Key Laboratory of Food Science and Health, School of Medicine, Nankai University, Tianjin 300350, Chinafuyousi@nankai.edu.cn (Y.F.);; 2 Research Institute of Public Health, Nankai University, Tianjin 300350, China; 3 Department of Chemical and Biochemical Engineering, College of Chemistry and Chemical Engineering, Xiamen University, Xiamen 361005, China; 4 Department of Clinical Laboratory, The Second Hospital of Tianjin Medical University, Tianjin 300211, China

**Keywords:** *Salmonella enterica*, bacteriophage-derived endolysin, turbidity reduction, lytic activity, polymyxin B, magnolol

## Abstract

Phage-derived endolysins are considered promising antibacterial agents for controlling bacterial pathogens. However, their activity against Gram-negative bacteria is usually limited by the outer membrane barrier. *Salmonella enterica* is a leading foodborne pathogen and an important target for endolysin-based control strategies. In this study, we identified and characterized LysSPYS2, an endolysin encoded by *Salmonella* phage SPYS_2. Recombinant LysSPYS2 was expressed in *Escherichia coli* Rosetta(DE3) and purified for activity assays. The optical density at 600 nm (OD_600_)-based turbidity-reduction activity, temperature and pH dependence under the tested conditions, and lytic spectrum of LysSPYS2 were determined using permeabilized bacterial suspensions. LysSPYS2 showed concentration-dependent turbidity reduction against *Salmonella enterica* serovar Typhimurium ATCC 14028, with the highest observed activity at 42 °C and pH 7. LysSPYS2 also showed lytic activity against several Gram-negative bacteria, with stronger activity against *Salmonella* strains. In intact-cell broth assays, LysSPYS2 alone showed limited growth suppression, whereas its growth-suppressive effect was enhanced when combined with polymyxin B or magnolol. These results suggest that LysSPYS2 may be further evaluated as a component of combination-based *Salmonella* control strategies.

## 1. Introduction

*Salmonella enterica* is one of the major foodborne pathogens threatening food safety and public health worldwide [1,2]. Consumption of contaminated food or water may cause salmonellosis, which is commonly characterized by diarrhea, fever, and abdominal cramps, and can lead to systemic infection in severe cases [1,2]. Due to its widespread distribution and increasing antimicrobial resistance, *Salmonella* has been listed as a priority pathogen by the World Health Organization (WHO) [3,4,5]. In China, *Salmonella* is also one of the predominant bacterial pathogens associated with acute diarrhea, emphasizing the need for effective strategies to control *Salmonella* contamination [4].

In the food industry, various approaches have been used to control *Salmonella* contamination, including thermal treatment, refrigeration, and chemical disinfectants [6]. However, these approaches may not completely eliminate contamination and may adversely affect the flavor, color, and nutritional quality of foods [1]. In addition, surviving *Salmonella* cells can form biofilms on food-processing surfaces, leading to persistent contamination during food production [7]. The increasing prevalence of multidrug-resistant *Salmonella* strains further complicates the control of this pathogen and highlights the need for alternative antimicrobial agents [8].

Phage-derived endolysins have attracted increasing attention as alternatives to conventional antimicrobial agents [9,10]. These enzymes are produced during the late stage of phage infection and can hydrolyze bacterial peptidoglycan, resulting in cell wall disruption and bacterial lysis [9,10]. Compared with conventional antibiotics, endolysins target conserved bacterial cell wall structures and have shown potential applications in controlling foodborne pathogens [10,11,12,13,14]. However, the application of endolysins against Gram-negative bacteria remains challenging because the outer membrane, which contains lipopolysaccharides (LPS), limits access to the peptidoglycan layer [15]. Therefore, strategies are needed to improve the activity of endolysins against Gram-negative bacteria. Previous studies have shown that membrane-disrupting agents, such as EDTA, can enhance the activity of *Salmonella*-targeting endolysins [10,16]. However, the potential toxicity and poor compatibility of these agents with food applications limit their practical use [17]. Polymyxin B is a last-resort antibiotic that disrupts the Gram-negative outer membrane by binding lipopolysaccharide [18,19]. Magnolol is a natural antibacterial neolignan from Magnolia officinalis that also perturbs bacterial membranes [20,21]. Both are therefore promising partners for enhancing endolysin activity against intact cells. Several *Salmonella* phage endolysins have been characterized to date, including LysSP1 [10] and rLysJNwz [9]; however, few studies have systematically evaluated their combined use with outer-membrane-disrupting agents (e.g., polymyxin B) or natural compounds (e.g., magnolol).

In this study, LysSPYS2, an endolysin encoded by *Salmonella* phage SPYS_2, was identified and characterized. Recombinant LysSPYS2 was expressed in *Escherichia coli* Rosetta(DE3) and purified. The OD_600_-based turbidity-reduction activity, apparent temperature and pH dependence, and lytic spectrum of LysSPYS2 were investigated using permeabilized bacterial suspensions. Furthermore, the combined effects of LysSPYS2 with polymyxin B and magnolol against intact *Salmonella enterica* serovar Typhimurium ATCC 14028 cells were evaluated. These findings provide a basis for assessing LysSPYS2 as a potential component in combination-based *Salmonella* control strategies.

## 2. Materials and Methods

### 2.1. Bacterial Strains and Culture Conditions

All bacterial strains used in this study are listed in Table 1. A total of 23 bacterial strains were used for the lytic-spectrum analysis, whereas *Escherichia coli* Rosetta(DE3) was used only for recombinant expression of LysSPYS2 and was not included in the lytic spectrum analysis. All bacterial strains were stored at −80 °C in LB broth containing 15% glycerol. Before each experiment, bacteria were streaked onto LB agar plates and incubated at 37 °C until colonies developed. A single colony was then inoculated into LB broth and cultured at 37 °C with shaking at 220 rpm for 16–22 h to obtain an overnight culture for subsequent experiments.

### 2.2. Cloning, Expression, and Purification of LysSPYS2

The coding sequence of LysSPYS2 was obtained from the genome sequence of *Salmonella* phage SPYS_2 [22]. A codon-optimized gene for expression in *Escherichia coli* was synthesized and cloned into the pET-28a vector between the BamHI and XhoI restriction sites. The recombinant plasmid was transformed into *E. coli *Rosetta(DE3) competent cells by chemical transformation, and transformants were selected on LB agar plates supplemented with kanamycin at 50 μg/mL.

For protein expression, a single colony was inoculated into LB broth supplemented with kanamycin at 50 μg/mL and incubated at 37 °C for 16–20 h. The overnight culture was transferred into fresh LB broth at a ratio of 1:100 and cultured at 37 °C until the OD_600_ reached 0.6–0.8. Protein expression was induced with 0.2 mM isopropyl β-D-1-thiogalactopyranoside (IPTG) at 30 °C for 16 h with shaking at 180 rpm. After induction, the cells were harvested by centrifugation, washed with phosphate-buffered saline (PBS), and resuspended in PBS at a ratio of 1 g wet cell pellet per 10 mL buffer. The cells were lysed by sonication at 220 W for 20 min using 10 s on/10 s off pulses. After centrifugation at 12,000 rpm and 4 °C for 60 min, the supernatant was collected for protein purification.

LysSPYS2 was purified by His-tag-based nickel affinity chromatography and eluted with 500 mM imidazole. The eluted protein was further purified by size-exclusion chromatography using an ÄKTA pure protein purification system (Cytiva, Uppsala, Sweden) with PBS as the running buffer. The purified LysSPYS2 protein was stored in PBS supplemented with 10% glycerol at −80 °C. The protein concentration was measured using a NanoDrop One spectrophotometer (Thermo Fisher Scientific, Waltham, MA, USA).

### 2.3. SDS-PAGE, Western Blotting, and Mass Spectrometry Identification of LysSPYS2

Purified LysSPYS2 was analyzed by 12% SDS-PAGE and stained with Coomassie brilliant blue. For Western blotting analysis, the protein was transferred to a membrane after SDS-PAGE. The membrane was probed with an anti-His primary antibody and a horseradish peroxidase (HRP)-conjugated anti-mouse secondary antibody. The signal was detected using enhanced chemiluminescence (ECL) reagent.

For mass spectrometry analysis, the target protein band was excised from the SDS-PAGE gel and subjected to mass spectrometric identification. The resulting peptides were matched against the amino acid sequence of LysSPYS2.

### 2.4. Bioinformatic Analysis of LysSPYS2

The amino acid sequence of LysSPYS2 was analyzed using several bioinformatic tools [23,24,25,26,27,28,29]. Conserved domains were predicted using InterPro and the NCBI Conserved Domain Database (CDD) [23,24]. Physicochemical properties, including molecular weight, theoretical isoelectric point (pI), instability index, aliphatic index, and grand average of hydropathicity (GRAVY), were calculated using the ProtParam tool (https://web.expasy.org/protparam/, accessed on 15 August 2026) on the ExPASy server [25]. Signal peptides were predicted using SignalP 6.0, and transmembrane helices were predicted using DeepTMHMM v1.0.57 (https://dtu.biolib.com/DeepTMHMM, accessed on 15 August 2026) [26,27]. The tertiary structure of LysSPYS2 was predicted using AlphaFold 3 and visualized using UCSF ChimeraX [28,29]. The LysSPYS2 amino acid sequence was searched against UniProtKB using BLASTP 2.16.0+ with the BLOSUM62 matrix. The search was performed on 24 August 2026. The 20 highest-ranked hits are listed in Supplementary Table S1.

### 2.5. OD_600_-Based Turbidity Reduction Assay in Permeabilized Bacterial Suspensions

A single colony of *S. Typhimurium* ATCC 14028 was inoculated into LB broth and cultured at 37 °C with shaking at 220 rpm for 16–20 h. The overnight culture was diluted 1:100 in fresh LB broth and incubated at 37 °C with shaking at 220 rpm for 4 h. The culture was then treated with 0.5% chloroform for 20 min. After treatment, the cells were collected by centrifugation at 5000× *g* and 4 °C for 10 min, washed with PBS, and resuspended in 50 mM Tris-HCl buffer (pH 8.2) supplemented with 0.1% Triton X-100.

LysSPYS2 was prepared in PBS and diluted to 1 mg/mL, followed by serial dilution to 0.1 mg/mL and 0.01 mg/mL. Then, 100 μL of each protein dilution was added to 5 mL of the bacterial suspension to obtain final concentrations of 20, 2, and 0.2 μg/mL, respectively. PBS was used as the control. The OD_600_ was measured every hour for 6 h, and additional measurements were taken at 18 and 24 h.

### 2.6. Effects of Reaction Temperature and pH on LysSPYS2 Activity

The effect of temperature on LysSPYS2 activity was assessed by monitoring OD_600_-based turbidity reduction under the indicated assay conditions. Briefly, *S. Typhimurium* ATCC 14028 cells were prepared as described above and resuspended in 50 mM Tris-HCl buffer (pH 8.2) supplemented with 0.1% Triton X-100. LysSPYS2 was diluted to 1 mg/mL in PBS, and 100 μL of the protein solution was added to 5 mL of bacterial suspension to obtain a final concentration of 20 μg/mL. PBS was used as the control. The reaction mixtures were incubated at 4, 25, 30, 37, 42, or 50 °C for 1 h, and OD_600_ was then measured to assess turbidity reduction.

The effect of pH on LysSPYS2 activity was assessed by incubating LysSPYS2 with bacterial suspensions under different pH conditions. Harvested *S. Typhimurium* ATCC 14028 cells were resuspended in 50 mM Tris-HCl buffer supplemented with 0.1% Triton X-100 at pH 5, 6, 7, 8, 9, or 10. LysSPYS2 was diluted to 1 mg/mL in PBS, and 10 μL of the protein solution was added to 0.5 mL of each bacterial suspension to obtain a final concentration of 20 μg/mL. The mixtures were incubated at 42 °C for 1 h, and PBS was used as the control. The OD_600_ was measured to assess turbidity reduction.

For both assays, relative turbidity reduction was calculated using the following formula: relative turbidity reduction = 1 − (ODt,T/ODt,C), where ODt,T and ODt,C represent the OD_600_ values of the treatment and control groups at the same time point, respectively.

### 2.7. Lytic Spectrum Analysis

The OD_600_-based lytic spectrum of LysSPYS2 was determined against the 23 bacterial strains listed in Table 1. Overnight cultures were prepared as described in Section 2.1. For Gram-negative bacteria, the cultures were treated with 0.5% chloroform for 20 min, whereas Gram-positive bacteria were not pretreated with chloroform. The cells were then collected by centrifugation at 5000× *g* and 4 °C for 20 min, washed with PBS, and resuspended in 50 mM Tris-HCl buffer (pH 8.2) supplemented with 0.1% Triton X-100.

LysSPYS2 was diluted to 1 mg/mL in PBS. Then, 10 μL of the protein solution was added to 0.5 mL of each bacterial suspension to obtain a final concentration of 20 μg/mL. PBS was used as the control. The mixtures were incubated at 42 °C for 1 h, and the OD_600_ was measured to determine turbidity activity. Relative turbidity reduction was calculated as 1 − (ODt,T/ODt,C), where ODt,T and ODt,C represent the OD_600_ values of the LysSPYS2-treated and control groups, respectively. The resulting values were visualized as a heatmap, and bacterial strains were hierarchically clustered and annotated according to genus.

### 2.8. Combined Growth-Suppressive Effect of LysSPYS2 and Polymyxin B Against Intact S. Typhimurium Cells

*S. Typhimurium* ATCC 14028 was streaked from a glycerol stock onto LB agar plates and cultured at 37 °C for 16–18 h. A single colony was inoculated into 5 mL LB broth and cultured at 37 °C with shaking at 220 rpm overnight. The overnight culture was then diluted 1:100 in fresh LB broth and cultured at 37 °C with shaking at 220 rpm until the mid-logarithmic phase was reached. Fresh stock solutions of polymyxin B sulfate (256 μg/mL) and LysSPYS2 (1 mg/mL) were prepared on ice before use.

The assay was performed in a 96-well plate. For the LysSPYS2 group, 100 μL bacterial suspension, 4 μL LysSPYS2 stock, and 96 μL LB broth were mixed to obtain a final LysSPYS2 concentration of 20 μg/mL. The total reaction volume in each well was 200 μL. For the polymyxin B group, 100 μL bacterial suspension was mixed with 100 μL LB broth containing polymyxin B at final concentrations of 0.25, 0.5, 1, 2, 4, 8, or 16 μg/mL. For the combined treatment group, 100 μL bacterial suspension, 4 μL LysSPYS2 stock, and 96 μL LB broth containing polymyxin B at the indicated concentrations were added. Bacterial suspension mixed with LB broth was used as the negative control.

The plate was incubated statically at 37 °C, and the OD_600_ was measured at 0, 1, 2, 3, 4, and 6 h.

### 2.9. Combined Effect of LysSPYS2 and Magnolol Against Intact S. Typhimurium Cells

Magnolol was prepared in dimethyl sulfoxide (DMSO) as a concentrated stock solution of 1024 μg/mL, and LysSPYS2 was prepared at 1 mg/mL. Working solutions of magnolol were prepared in LB broth with matched DMSO content so that the final DMSO concentration was the same across all relevant groups. The total reaction volume in each well was 200 μL.

The assay was performed in a 96-well plate. For the LysSPYS2 group, 100 μL bacterial suspension, 4 μL LysSPYS2 stock, and 96 μL LB broth were mixed to obtain a final LysSPYS2 concentration of 20 μg/mL. For the magnolol group, 100 μL bacterial suspension was mixed with 100 μL magnolol working solution at final concentrations of 1, 2, 4, 8, 16, 32, 64, or 128 μg/mL. For the combined treatment group, 100 μL bacterial suspension, 4 μL LysSPYS2 stock, and 96 μL LB broth containing the indicated concentrations of magnolol were added. LB broth supplemented with matched DMSO was used as the vehicle control, and bacterial suspension mixed with LB broth was used as the negative control.

The plate was incubated statically at 4, 25, 37, or 42 °C, and the OD_600_ was measured after 24 h.

### 2.10. Statistical Analysis

All experiments were performed with at least three biological replicates, and the data are presented as means ± SD. Statistical analysis was performed using R software version 4.5.1. Differences among groups were assessed using one-way analysis of variance (ANOVA) followed by Tukey’s multiple comparisons test. A *p* value < 0.05 was considered statistically significant. Significant differences are indicated by different lowercase letters in the figures.

## 3. Results

### 3.1. Expression, Purification, and Characterization of Recombinant LysSPYS2

**Figure 1:**
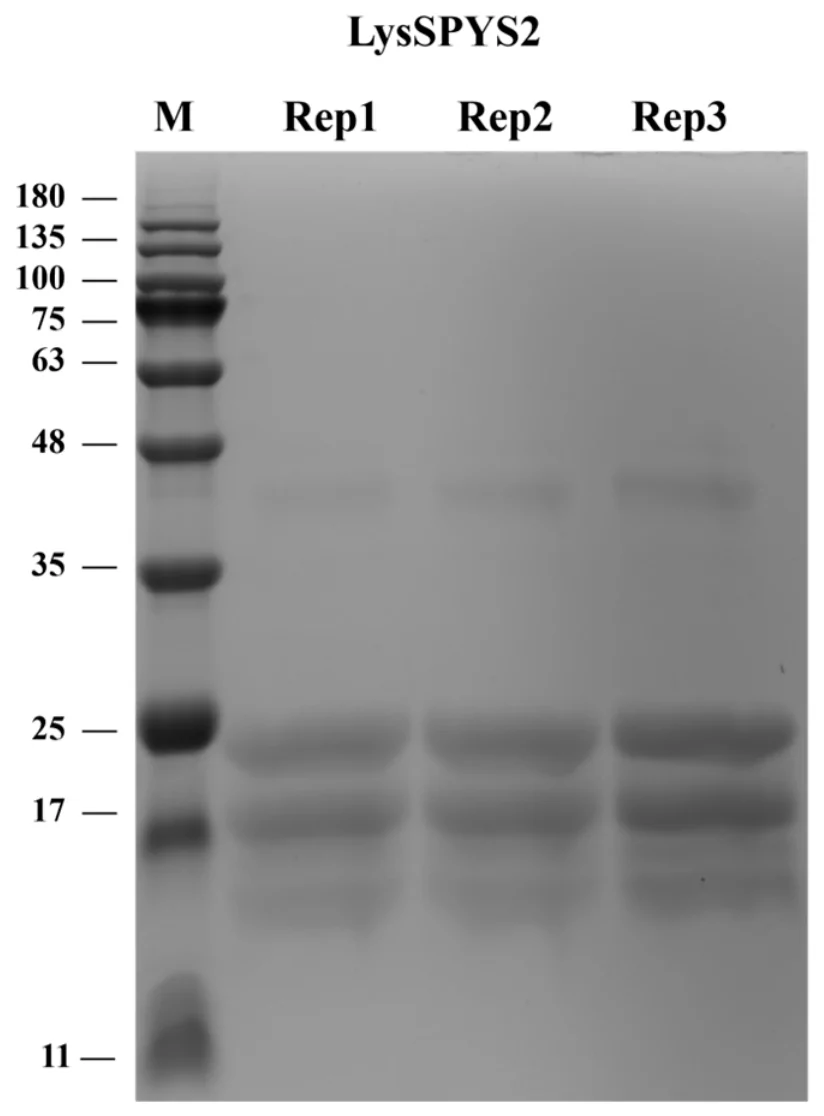
Western blot identification of recombinant LysSPYS2. Purified recombinant LysSPYS2 expressed in *Escherichia coli* Rosetta(DE3) was detected using an anti-His monoclonal antibody. Lane M, prestained protein molecular weight marker; lanes Rep1–Rep3, three replicate loadings of the purified LysSPYS2 sample. Molecular masses are indicated in kDa.

Recombinant LysSPYS2 was successfully expressed and purified using the *Escherichia coli* Rosetta(DE3) expression system. Western blot analysis using an anti-His antibody detected a band corresponding to the expected molecular mass of His-tagged LysSPYS2 (Figure 1). To further verify the identity of the purified protein, mass spectrometry analysis was performed. The identified peptide fragments matched the predicted amino acid sequence of LysSPYS2 with 91% sequence coverage, supporting the identity of the purified protein as recombinant LysSPYS2.

To further characterize LysSPYS2, its amino acid sequence, physicochemical properties, and predicted structure were analyzed using bioinformatic tools. LysSPYS2 contains 151 amino acid residues, with a theoretical molecular mass of 16.88 kDa. Conserved domain analysis using InterPro and the NCBI Conserved Domain Database suggested that LysSPYS2 is an amidase-like endolysin belonging to the amidase/PGRP superfamily. No recognizable cell wall-binding domain was detected, indicating that LysSPYS2 may mainly consist of a catalytic domain.

**Figure 2:**
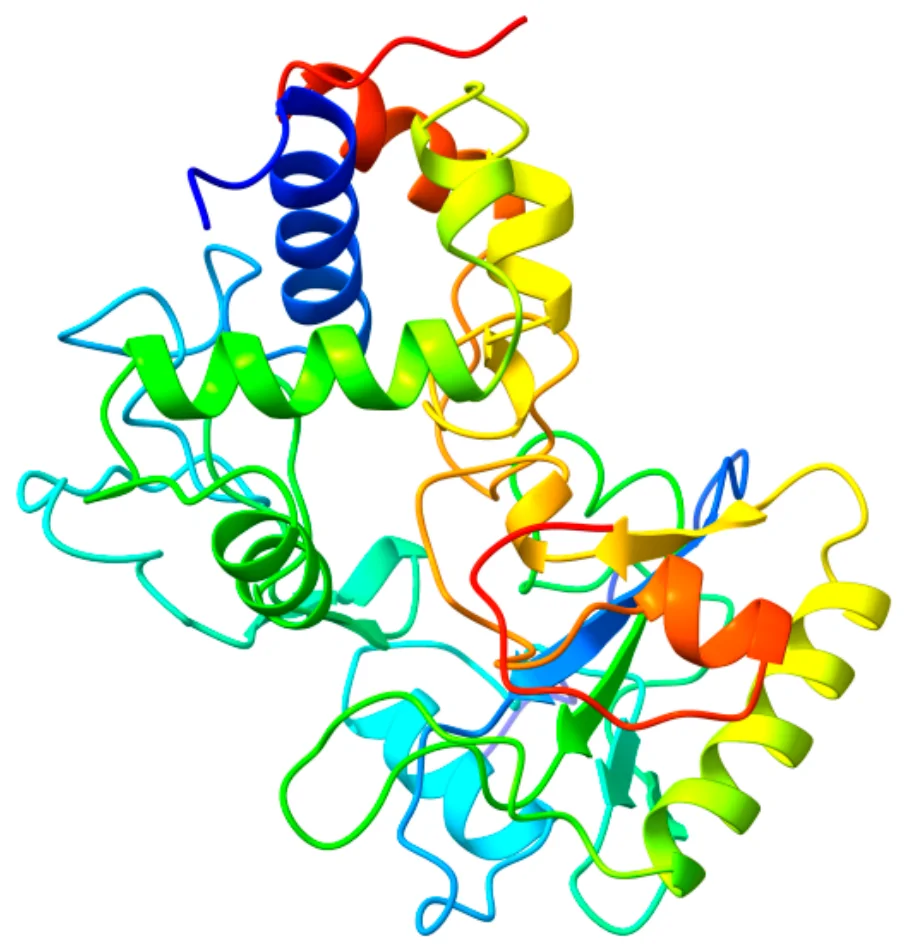
Predicted three-dimensional structure of LysSPYS2. The three-dimensional structure of LysSPYS2 was predicted from its amino acid sequence using AlphaFold 3 and visualized using UCSF ChimeraX version 1.12. The structure is shown in cartoon representation and colored from the N-terminus (blue) to the C-terminus (red).

ProtParam analysis predicted a theoretical isoelectric point (pI) of 7.05, an instability index of 43.07, an aliphatic index of 77.42, and a grand average of hydropathicity (GRAVY) value of −0.437. These results suggested that LysSPYS2 is a hydrophilic protein with a near-neutral theoretical pI. SignalP 6.0 predicted no signal peptide, and DeepTMHMM identified no transmembrane helix. Structural prediction using AlphaFold 3 revealed a compact globular architecture, consistent with the structural features commonly observed in amidase-type endolysins (Figure 2). The top 20 UniProtKB BLASTP hits were all annotated as endolysins from phages associated with Gram-negative bacterial hosts (Table S1), and 12 of them were associated with *Salmonella* or *Escherichia* phages.

### 3.2. OD_600_-Based Lytic Activity of LysSPYS2 Against Permeabilized S. Typhimurium

**Figure 3:**
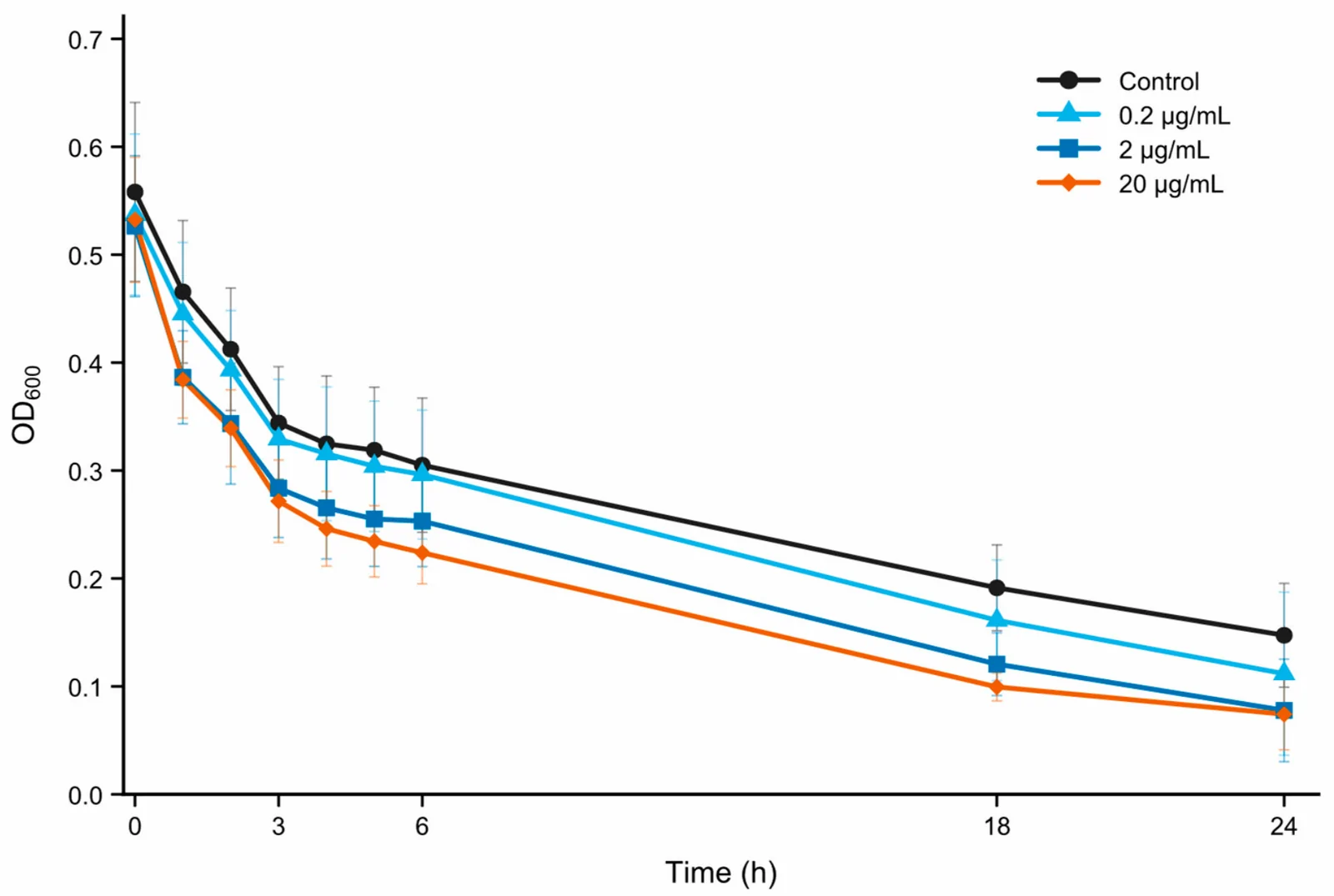
Concentration-dependent turbidity-reducing activity of LysSPYS2 against permeabilized *S. Typhimurium* ATCC 14028 suspensions. Bacterial suspensions were treated with LysSPYS2 at final concentrations of 0.2, 2, or 20 μg/mL, with PBS-treated bacteria used as the control. OD_600_ values were monitored at the indicated time points. Data are presented as mean ± SD from three independent experiments (*n* = 3).

The OD_600_-based turbidity-reducing activity of LysSPYS2 against permeabilized *S. Typhimurium* ATCC 14028 suspensions was evaluated by monitoring OD_600_ during incubation. LysSPYS2 treatment reduced turbidity in a concentration-dependent manner (Figure 3). Among the tested concentrations, 20 μg/mL LysSPYS2 showed the strongest reduction, with OD_600_ values continuously decreasing during the incubation period.

After 24 h of incubation, treatment with 0.2, 2, and 20 μg/mL LysSPYS2 reduced the OD_600_ values by approximately 24.2%, 47.2%, and 49.6%, respectively, compared with the untreated control. These results demonstrated that LysSPYS2 exhibits concentration-dependent turbidity-reducing activity against permeabilized *S. Typhimurium* ATCC 14028 suspensions under the assay conditions used in this study.

**Figure 4:**
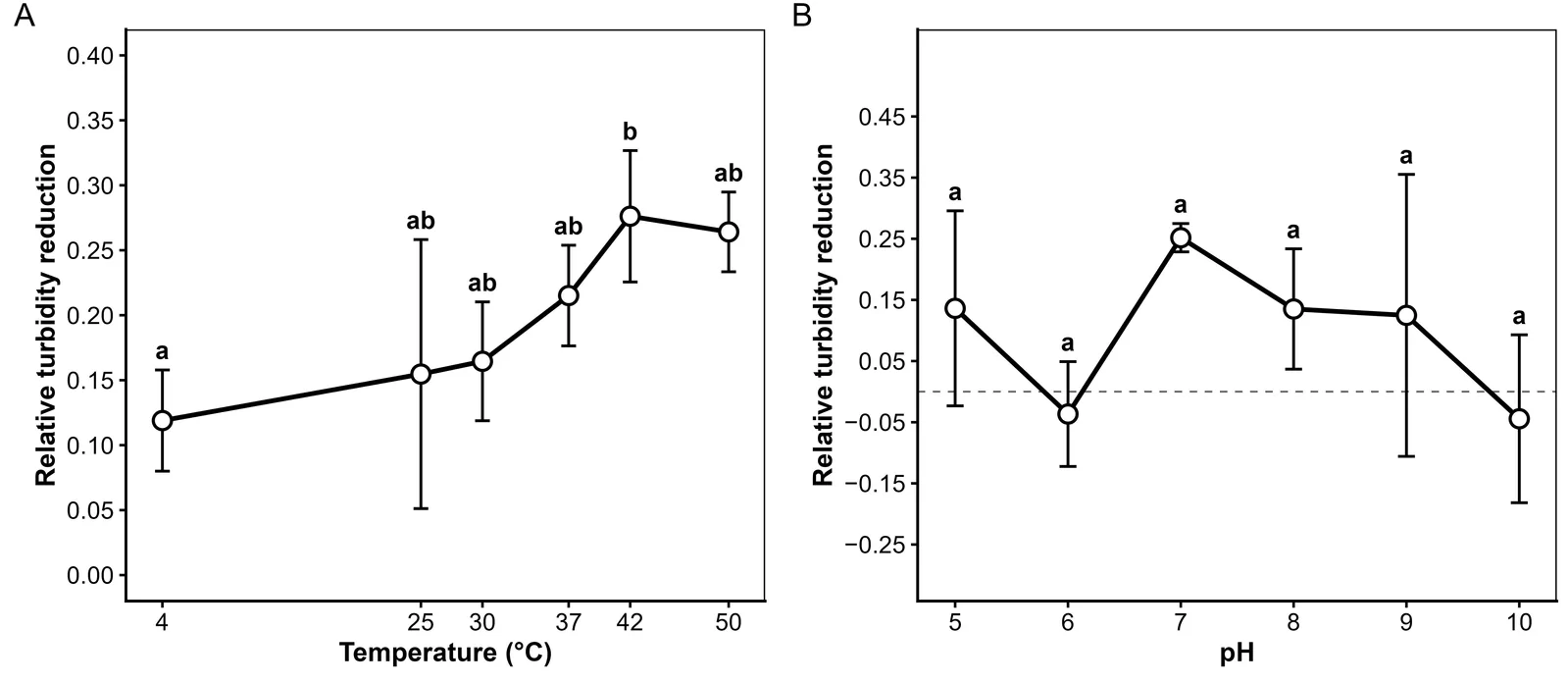
Effects of temperature and pH on the OD_600_-based lytic activity of LysSPYS2 against permeabilized *S. Typhimurium* ATCC 14028 suspensions. (**A**) Effect of temperature on LysSPYS2 activity. Bacterial suspensions were treated with LysSPYS2 and incubated at different temperatures (4, 25, 30, 37, 42, and 50 °C) for 1 h. (**B**) Effect of pH on LysSPYS2 activity. LysSPYS2 activity was determined under pH conditions ranging from 5 to 10 after incubation for 1 h. The lytic activity was calculated based on OD_600_ reduction. Data are presented as mean ± SD. Different lowercase letters indicate significant differences among groups (one-way ANOVA followed by Tukey’s post hoc test, *p* < 0.05); groups sharing a letter are not significantly different.

The effects of temperature and pH on LysSPYS2 activity were assessed by monitoring OD_600_-based lytic activity under different environmental conditions. LysSPYS2 retained detectable activity across the tested temperature range from 4 °C to 50 °C (Figure 4A). The highest activity was observed at 42 °C under the tested conditions, with a relative lysis value of 0.276, which was significantly higher than that at 4 °C (*p* < 0.05) but did not differ significantly from the other tested temperatures. Although the activity slightly decreased at 50 °C, LysSPYS2 still maintained measurable activity under this condition.

The pH dependence of LysSPYS2 was examined over a pH range of 5 to 10 (Figure 4B). The highest activity was observed at pH 7, with a relative turbidity reduction value of 0.252, although no significant differences were detected among the tested pH values (*p* > 0.05). In comparison, lower activity was observed at the other tested pH values, with little or no turbidity reduction at pH 6 and pH 10. These results suggest that near-neutral conditions are more favorable for LysSPYS2 activity, although the differences among pH values did not reach statistical significance.

### 3.3. Lytic Spectrum of LysSPYS2 Against Different Bacterial Strains

**Figure 5:**
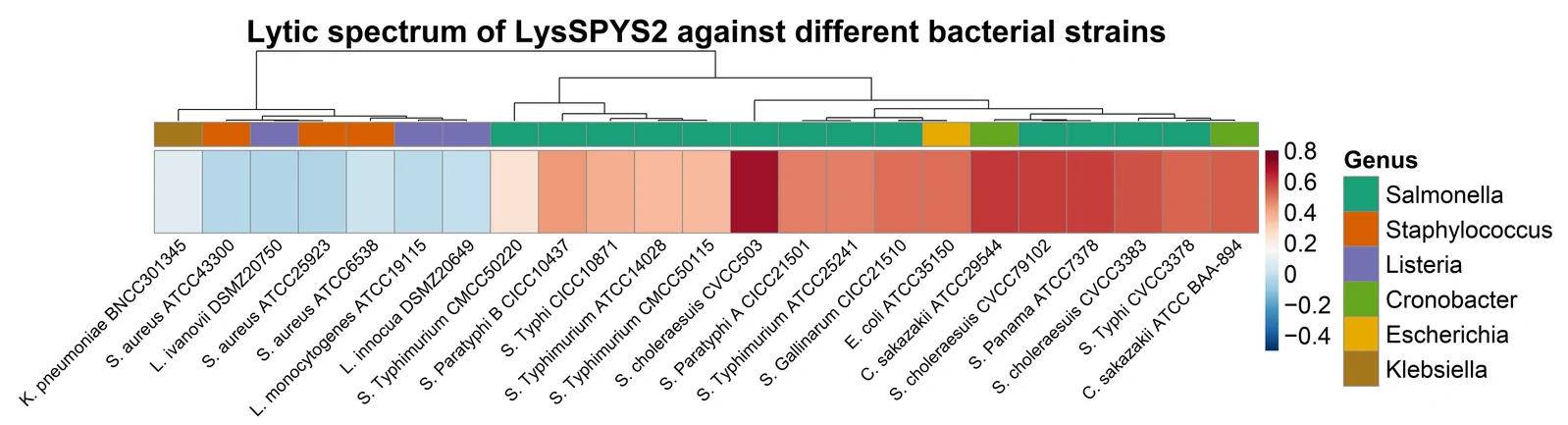
Lytic spectrum of LysSPYS2 against different bacterial strains. The OD_600_-based lytic spectrum of LysSPYS2 was evaluated against 23 bacterial strains, including Gram-negative and Gram-positive bacteria. Gram-negative bacteria were pretreated with 0.5% chloroform, whereas Gram-positive bacteria were not pretreated. LysSPYS2 was used at a final concentration of 20 μg/mL, and the reactions were incubated at 42 °C for 1 h. The heatmap shows relative turbidity reduction values, with higher values indicating stronger turbidity-reducing activity. Bacterial strains were hierarchically clustered and annotated according to genus.

The lytic spectrum of LysSPYS2 was evaluated against 23 Gram-negative and Gram-positive bacterial strains (Table 1 and Figure 5). LysSPYS2 showed preferential activity against *Salmonella* strains, with relative turbidity reduction values ranging from 0.24 to 0.70 among the tested strains. The highest activity was observed against *S. enterica* serovar Choleraesuis CVCC 503, with a relative turbidity reduction value of 0.697, followed by *S. enterica* serovar Panama ATCC 7378 and *S. enterica* serovar Choleraesuis CVCC 79102, with values of 0.597 and 0.593, respectively. Several *S. Typhimurium* strains, including ATCC 14028, ATCC 25241, CMCC 50220, and CMCC 50115, were also susceptible to LysSPYS2, with relative turbidity reduction values ranging from 0.24 to 0.48.

In addition to *Salmonella*, LysSPYS2 retained activity against several non-Salmonella Gram-negative bacteria. Among these strains, *Cronobacter sakazakii* ATCC BAA-894 and ATCC 29544 and *Escherichia coli* ATCC 35150 showed relatively high susceptibility, with relative turbidity reduction values above 0.50. In contrast, limited or negligible activity was detected against the tested Gram-positive bacteria, including *Listeria* spp. and *Staphylococcus aureus*. These results suggested that LysSPYS2 preferentially acted on *Salmonella* strains while retaining activity against selected non-Salmonella Gram-negative bacteria under the permeabilized-cell assay conditions. Negative relative turbidity reduction values indicate that the OD_600_ of treated samples slightly exceeded that of the control, likely owing to bacterial growth or settling during the 1-h incubation, and were interpreted as no lytic activity.

### 3.4. Combined Growth-Suppressive Effect of LysSPYS2 and Polymyxin B Against S. Typhimurium

**Figure 6:**
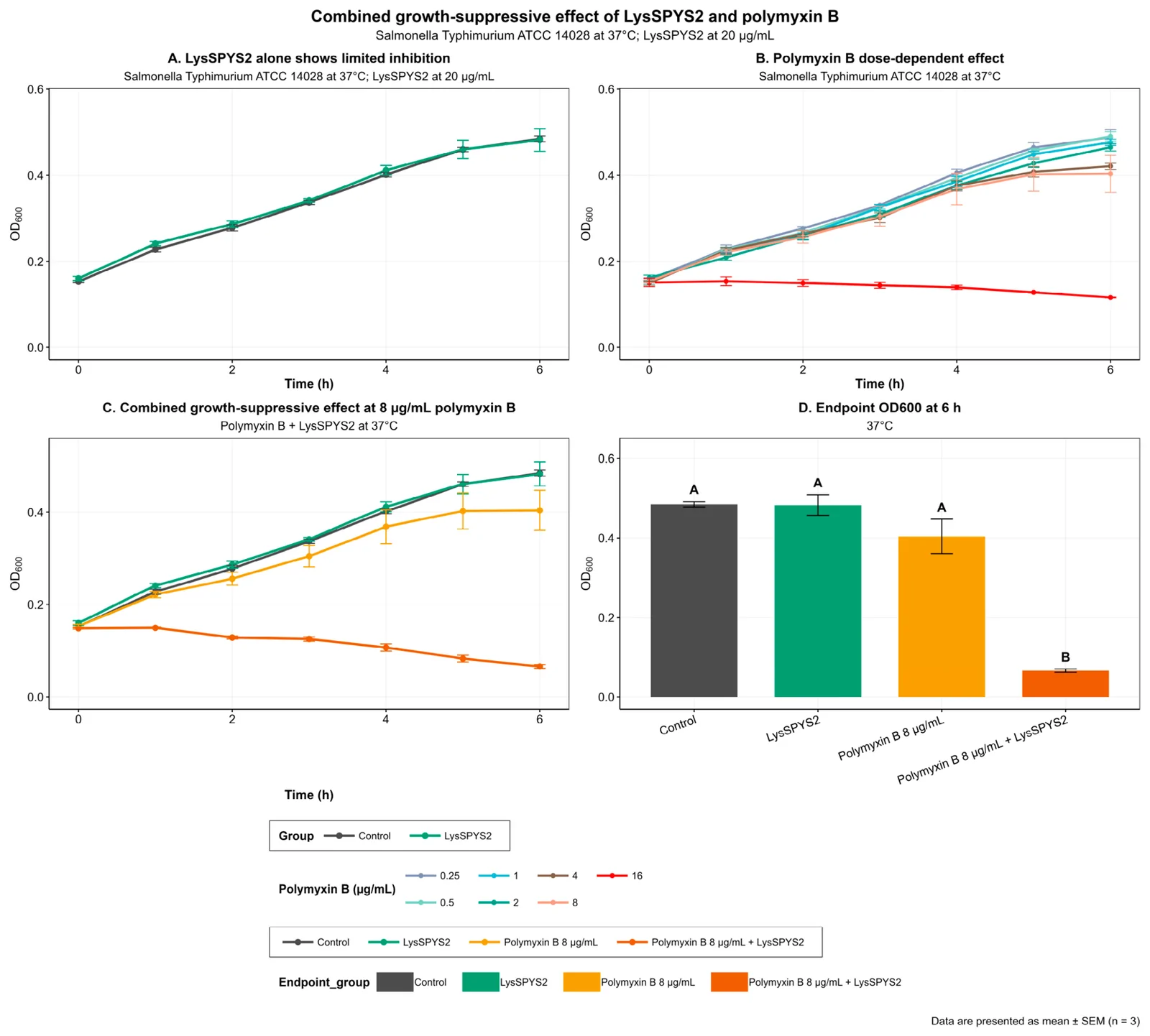
Combined growth-suppressive effect of LysSPYS2 and polymyxin B against *S. Typhimurium* ATCC 14028. (**A**) Growth curves of the control and LysSPYS2-alone groups. (**B**) Dose-dependent growth inhibition by polymyxin B alone. (**C**) Growth curves of polymyxin B (8 μg/mL) alone or combined with LysSPYS2. (**D**) Endpoint OD_600_ values at 6 h for the indicated groups. Different letters indicate significant differences based on one-way ANOVA followed by Tukey’s post hoc test (*p* < 0.05). Data are presented as mean ± SD (*n* = 3).

The combined growth-suppressive effect of LysSPYS2 and polymyxin B against intact *S. Typhimurium* ATCC 14028 cells was evaluated by monitoring OD_600_ during a 6 h incubation period (Figure S1). LysSPYS2 alone showed only a limited growth-suppressive effect under the tested conditions (Figure 6A). In comparison, polymyxin B displayed a dose-dependent inhibitory effect, with higher concentrations resulting in progressively lower OD_600_ values (Figure 6B).

When LysSPYS2 was combined with polymyxin B, the OD_600_ values were lower than those observed with polymyxin B alone. At 8 μg/mL polymyxin B, the combination treatment consistently resulted in lower OD_600_ values throughout the incubation period than polymyxin B alone (Figure 6C). Endpoint analysis at 6 h also showed lower OD_600_ values in the combination treatment group than in the corresponding single-treatment groups (Figure 6D). These results indicated that the combination of LysSPYS2 and polymyxin B produced a greater OD_600_-based growth-suppressive effect against *S. Typhimurium* ATCC 14028 than either treatment alone under the tested conditions.

### 3.5. Combined Effect of Magnolol and LysSPYS2 Against S. Typhimurium

The combined effect of magnolol and LysSPYS2 against intact *S. Typhimurium* ATCC 14028 cells was evaluated after 24 h of incubation at 4, 25, 37, and 42 °C. OD_600_ values were visualized using heatmaps to compare the effects of magnolol alone and magnolol combined with LysSPYS2. In the magnolol-alone group, 0 μg/mL represented the matched vehicle control, whereas in the combination group, 0 μg/mL represented LysSPYS2 alone.

**Figure 7:**
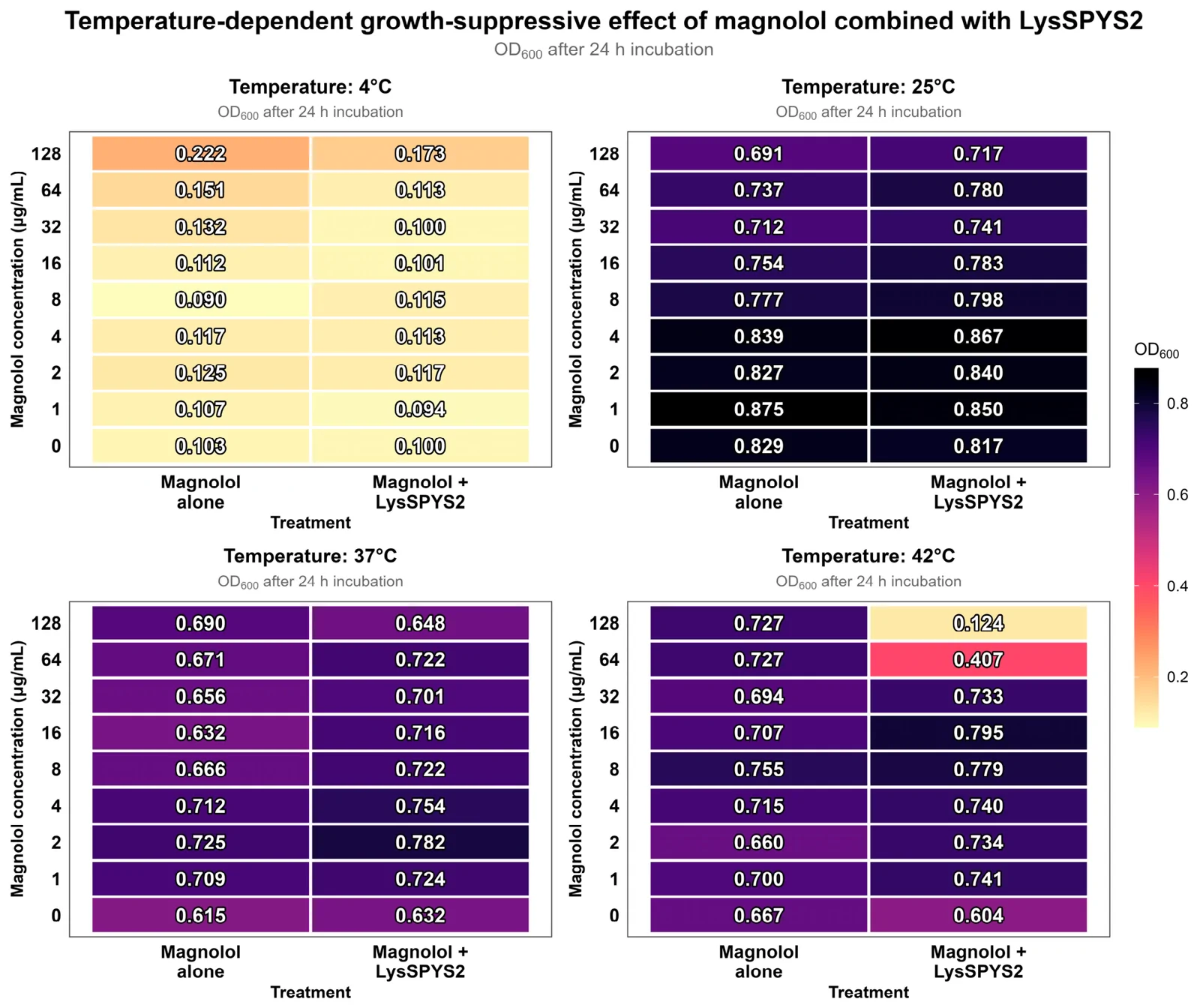
Temperature-dependent combined effect of magnolol and LysSPYS2 against *S. Typhimurium* ATCC 14028. Heatmaps show OD_600_ values after 24 h incubation at 4, 25, 37, and 42 °C. The *y*-axis indicates the concentration of magnolol. In the “Magnolol alone” group, 0 μg/mL represents the matched vehicle control, whereas 1–128 μg/mL represent magnolol treatment alone. In the “Magnolol + LysSPYS2” group, 0 μg/mL represents LysSPYS2 treatment alone, whereas 1–128 μg/mL represent combined treatment with magnolol and LysSPYS2. Lower OD_600_ values indicate stronger growth suppression under the tested conditions.

The OD_600_ heatmap showed that the effect of magnolol varied with both concentration and temperature (Figure 7 and Figure S2). At 4 °C, bacterial growth remained low across most treatment conditions, and differences among treatments were limited. At 25 °C and 37 °C, magnolol alone showed a concentration-dependent reduction in OD_600_, whereas the addition of LysSPYS2 did not substantially change the endpoint OD_600_ values.

At 42 °C, the combination of magnolol and LysSPYS2 produced lower OD_600_ values at higher magnolol concentrations than magnolol alone. These observations suggested a temperature- and concentration-dependent combined effect under the tested conditions, although further confirmation using viability-based assays is required.

## 4. Discussion

Phage-derived endolysins have attracted increasing attention as antibacterial agents because they can directly hydrolyze bacterial peptidoglycan. However, their external application against Gram-negative bacteria remains challenging, as the outer membrane prevents endolysins from reaching the peptidoglycan layer [17,30]. This limitation is particularly important for the control of foodborne Gram-negative pathogens such as *Salmonella*.

In our previous study, the lytic *Salmonella* phage SPYS_2 was isolated, characterized, and sequenced [22]. The present study extends this work by identifying and characterizing LysSPYS2, an endolysin encoded by phage SPYS_2. LysSPYS2 was successfully expressed and purified, and mass spectrometry confirmed the identity of the recombinant protein. Bioinformatic analysis showed that LysSPYS2 belongs to the amidase/PGRP superfamily, suggesting that it may hydrolyze specific bonds within bacterial peptidoglycan and thereby disrupt cell-wall integrity [31,32]. Compared with previously reported Salmonella endolysins such as LysSP1, rLysJNwz, Lys68, and Salmcide-p1 [9,10,33,34], LysSPYS2 originates from our newly isolated phage SPYS_2, and its combined activity with polymyxin B and magnolol has not been reported previously. LysSPYS2 is a relatively small protein, and no obvious cell wall-binding domain, signal peptide, or transmembrane helix was predicted. This architecture is consistent with many endolysins from Gram-negative phages, which are often mainly composed of a catalytic domain [17,30,35,36].

LysSPYS2 showed concentration-dependent OD_600_-based turbidity reduction against *S. Typhimurium* ATCC 14028. However, in the initial activity, temperature, pH, and lytic-spectrum assays, Gram-negative cells were treated with chloroform and Triton X-100, which could compromise the bacterial envelope and facilitate enzyme access to peptidoglycan. Therefore, these assays mainly demonstrated the intrinsic muralytic activity of LysSPYS2 after the peptidoglycan layer became accessible, rather than its ability to independently penetrate the outer membrane of intact Gram-negative cells. Likewise, the intact-cell combination assays monitored OD_600_-based growth suppression rather than bacterial killing, and therefore should not be interpreted as direct evidence of bactericidal activity.

The activity of LysSPYS2 was affected by temperature and pH. LysSPYS2 retained detectable lytic activity from 4 °C to 50 °C and showed the highest activity at 42 °C under the tested conditions. This higher activity at 42 °C may reflect the optimal lysis temperature of the parental phage SPYS_2 and a thermostable conformational state of the enzyme, although further characterization is warranted. It also showed the highest activity at pH 7, suggesting that near-neutral conditions are more favorable, although the differences among pH values were not statistically significant. Similar pH preferences and thermal tolerances have been reported for other *Salmonella* phage endolysins, such as Lys68 and Salmcide-p1 [33,34]. These characteristics may be useful for further evaluation of LysSPYS2 as an antibacterial candidate.

The lytic-spectrum assay showed that LysSPYS2 was active against several *Salmonella* serovars and strains, and activity was also observed against selected non-Salmonella Gram-negative bacteria, including *E. coli* and *Cronobacter sakazakii*. This activity pattern was broader than the host range of the parental phage, which is reasonable because productive phage infection depends on receptor recognition, adsorption, genome entry, and intracellular replication, whereas purified endolysin activity mainly depends on peptidoglycan accessibility and substrate compatibility [37,38]. Similar differences between the host range of phages and the activity spectrum of their endolysins have been reported previously [39,40]. The top 20 BLASTP homologues were all annotated as endolysins from phages associated with Gram-negative bacterial hosts (Supplementary Table S1), which was broadly consistent with the activity pattern observed in the lytic-spectrum assay. However, the lytic activity varied among strains, which may be related to differences in cell-envelope structure, peptidoglycan accessibility, or assay conditions.

When tested against intact *S. Typhimurium* cells, LysSPYS2 alone showed only limited growth-suppressive activity. This result is consistent with the outer membrane barrier of Gram-negative bacteria. A conventional minimum inhibitory concentration (MIC) of LysSPYS2 alone against intact cells is difficult to define; we therefore evaluated its effect in combination with the membrane-permeabilizing agent polymyxin B. Polymyxin B can interact with lipopolysaccharide and disrupt the outer membrane of Gram-negative bacteria by displacing the divalent cations (Mg^2+^ and Ca^2+^) that stabilize the lipopolysaccharide layer [18,19,41]. The MIC of polymyxin B against *S. Typhimurium* ATCC 14028 has been reported to be 1.25 μg/mL [42], and the polymyxin B concentrations used here (0.25–16 μg/mL) were selected to span this MIC. Under our growth-suppression conditions, 8 μg/mL polymyxin B alone was sub-inhibitory, whereas its combination with LysSPYS2 markedly suppressed growth. This higher apparent sub-inhibitory level reflects our use of log-phase, high-density cells and a 6-h growth readout, which differ from the standard MIC conditions. In this study, the combination of LysSPYS2 and polymyxin B resulted in stronger OD_600_-based growth suppression than either treatment alone. This enhanced effect may be due to increased access of LysSPYS2 to the peptidoglycan layer after outer membrane perturbation by polymyxin B [43,44].

Magnolol was further evaluated as a potential antibacterial partner for LysSPYS2. Magnolol, a bioactive neolignan isolated from Magnolia officinalis, has been reported to exhibit antibacterial activity against multiple bacterial species [20,21,45]. In contrast to polymyxin B, magnolol may affect multiple cellular targets, and its antibacterial mechanism can vary among bacteria. Therefore, the magnolol experiment should not be interpreted simply as another outer membrane-permeabilization assay. The combined effect of LysSPYS2 and magnolol varied with temperature and was most evident at 42 °C under the tested conditions. This temperature-restricted enhancement may be attributed not only to the higher catalytic activity of LysSPYS2 at 42 °C but also to increased outer-membrane fluidity and membrane stress in *Salmonella* at elevated temperature, which could facilitate magnolol-mediated membrane perturbation and subsequent access of the enzyme to peptidoglycan. Nevertheless, the requirement for an elevated temperature limits the practical utility of this combination in clinical and food-storage settings, which typically operate at lower temperatures; therefore, this finding should be regarded as a proof-of-concept rather than a directly applicable strategy. The observed effect was also mainly associated with higher magnolol concentrations and requires confirmation using viable-cell counts or time-kill assays.

Overall, this study identified and characterized LysSPYS2 as an amidase-like endolysin encoded by *Salmonella* phage SPYS_2, and showed that its growth-suppressive activity could be enhanced by polymyxin B or magnolol under selected conditions. While these findings support the potential of LysSPYS2 in combination-based antibacterial strategies, further work is needed to confirm its activity in viable-cell assays, clarify the mechanisms underlying the observed combined effects, and evaluate its performance against biofilms formed by foodborne *Salmonella* and in food-related systems.

## 5. Conclusions

In conclusion, this study characterized LysSPYS2, an amidase-like endolysin encoded by *Salmonella* phage SPYS_2. LysSPYS2 exhibited muralytic activity in permeabilized-cell assays and enhanced OD_600_-based growth suppression of intact *S. Typhimurium* when combined with polymyxin B, whereas magnolol showed a weaker, temperature-dependent combined effect. However, direct bactericidal activity against intact cells remains to be confirmed by viable-cell assays. These findings support the further evaluation of LysSPYS2 as a candidate component of combination-based *Salmonella* control strategies.

## Figures and Tables

**Table 1:** Bacterial strains used in this study.

Strain ID Number	Species	Serovar (*Salmonella* Only)
ATCC 14028 ^a^	*Salmonella enterica*	Typhimurium
CMCC 50115 ^b^	*Salmonella enterica*	Typhimurium
CMCC 50220	*Salmonella enterica*	Typhimurium
ATCC 25241	*Salmonella enterica*	Typhimurium
CICC 10437 ^c^	*Salmonella enterica*	Paratyphi B
CICC 21501	*Salmonella enterica*	Paratyphi A
CICC 10871	*Salmonella enterica*	Typhi
CVCC 3378 ^d^	*Salmonella enterica*	Typhi
CICC 21510	*Salmonella enterica*	Gallinarum
CVCC 3383	*Salmonella enterica*	Choleraesuis
CVCC 503	*Salmonella enterica*	Choleraesuis
CVCC 79102	*Salmonella enterica*	Choleraesuis
ATCC 7378	*Salmonella enterica*	Panama
ATCC 35150	*Escherichia coli*	NA ^g^
ATCC BAA-894	*Cronobacter sakazakii*	NA
ATCC 29544	*Cronobacter sakazakii*	NA
BNCC 301345 ^e^	*Klebsiella pneumoniae*	NA
ATCC 19115	*Listeria monocytogenes*	NA
DSMZ 20750 ^f^	*Listeria ivanovii*	NA
DSMZ 20649	*Listeria innocua*	NA
ATCC 25923	*Staphylococcus aureus*	NA
ATCC 43300	*Staphylococcus aureus*	NA
ATCC 6538	*Staphylococcus aureus* subsp. *aureus*	NA

^a^ ATCC stands for the American Type Culture Collection (https://www.atcc.org/, accessed on 18 August 2026); ^b^ CMCC stands for National Center for Medical Culture Collections (https://www.cmccb.org.cn, accessed on 18 August 2026); ^c^ CICC stands for China Center of Industrial Culture Collection (http://english.china-cicc.org, accessed on 18 August 2026); ^d^ CVCC stands for National Center for Veterinary Culture Collections (http://cvcc.ivdc.org.cn, accessed on 18 August 2026). ^e^ BNCC stands for BeNa Culture Collection (https://www.bncc.com, accessed on 8 August 2026). ^f^ DSMZ stands for German Collection of Microorganisms and Cell Cultures GmbH (https://www.dsmz.de, accessed on 8 August 2026). ^g^ NA, not applicable to the Salmonella-only serovar field; no serovar information is reported for the non-Salmonella strains in this table.

## Data Availability

The original contributions presented in this study are included in the article/Supplementary Material. Further inquiries can be directed to the corresponding author.

## Supplementary Materials

The following supporting information can be downloaded at: https://www.mdpi.com/article/10.3390/microorganisms14102407/s1, Figure S1: Time-course growth curves of *S. Typhimurium* ATCC 14028 treated with polymyxin B alone or combined with LysSPYS2; Figure S2: Temperature-dependent growth inhibition rates of magnolol combined with LysSPYS2 against *S. Typhimurium* ATCC 14028; Table S1: Top 20 UniProtKB BLASTP hits for the native LysSPYS2 sequence.
