# Supplementary material for: Characterization of a Salmonella Phage-Derived Endolysin LysSPYS2 and Its Combined Activity with Polymyxin B and Magnolol
Source: Microorganisms. 2026 Oct 21;14(10):2407. doi: 10.3390/microorganisms14102407 (PMC13707791; doi:10.3390/microorganisms14102407)
Supplement: Supplementary file 1 [file microorganisms-14-02407-s001.zip › microorganisms-4610767-supplementary.pdf]

Table S1. Top 20 UniProtKB BLASTP hits for the native LysSPYS2 sequence.

Rank	UniProt accession	Phage / organism	Identity	E-value
1	A0A9E7T687	*Salmonella* phage vB_SalM_PC127	149/151 (98.7%)	2.4e-108
2	U5P3N2	*Erwinia* phage FE44	147/151 (97.4%)	2.3e-106
3	A0A3G1L2Y1	*Salmonella* phage BSP161	144/151 (95.4%)	2.7e-105
4	A0AAE7TQ68	*Pectobacterium* phage PcCB251	140/151 (92.7%)	3.1e-104
5	A0ABZ2BLS4	*Escherichia* phage P151	144/151 (95.4%)	4.5e-104
6	E5L7D0	*Yersinia* phage Yep-phi	138/151 (91.4%)	3.5e-101
7	A0A1J0MEU6	*Pectobacterium* phage PP74	140/151 (92.7%)	1.4e-100
8	A0A7G3WWN7	*Escherichia* phage PhiV-1	137/151 (90.7%)	2.9e-100
9	A0A6M2Z6W9	*Salmonella* phage vB_SalM-LPST153	135/151 (89.4%)	5.9e-100
10	A0AAE9NJP9	*Salmonella* phage JSS2	135/151 (89.4%)	5.9e-100
11	B6Z9F7	*Kluyvera* phage Kvp1	136/151 (90.1%)	8.3e-100
12	A0A4D6DRQ9	*Shigella* phage VB_Ship_A7	137/151 (90.7%)	8.3e-100
13	A0A7G5BCT8	*Enterobacter* phage PZJ0206	136/151 (90.1%)	1.7e-99
14	D3W693	*Escherichia* phage 285P	135/151 (89.4%)	2.4e-99
15	A0AAE9NMP1	*Salmonella* phage JSS1	135/151 (89.4%)	3.4e-99
16	A0AA51N3Y8	*Escherichia* phage pO103	136/151 (90.1%)	4.8e-99
17	A0A2Z4Q2C1	*Escherichia* phage vB_EcoP_S523	135/151 (89.4%)	9.7e-99
18	A0AA96SIQ0	*Salmonella* phage SPLA4	135/151 (89.4%)	9.7e-99
19	A0AAE8AZB1	*Escherichia* phage ErnstBeyeler	134/151 (88.7%)	9.7e-99
20	A0A2D0PEC9	*Yersinia* phage fPS-59	132/151 (87.4%)	2.0e-98

Note: The search was performed against UniProtKB on 24 August 2026 (BLASTP 2.16.0+; BLOSUM62; UniProt BLAST Job ID: ncbiblast-R20260824-033849-0187-1111797-p2m). All Top 20 entries were annotated as endolysins and were unreviewed TrEMBL records. All aligned subjects were 151 aa, with 100.0% query coverage, a 151-aa alignment, and zero gaps. E-values are from the TSV export. Database annotations were not treated as independent experimental validation.

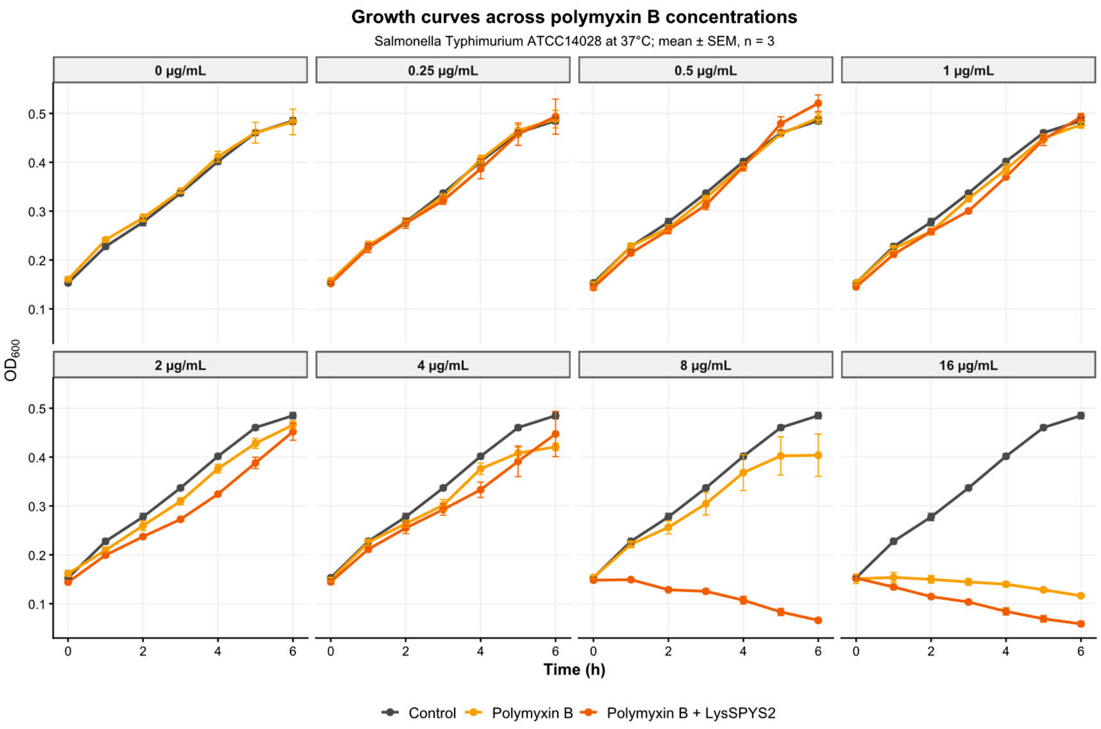

Figure S1. Time-course growth curves of *S. Typhimurium* ATCC 14028 treated with polymyxin B alone or in combination with LysSPYS2.

Bacterial suspensions were exposed to polymyxin B at 0.25, 0.5, 1, 2, 4, 8, or 16 μg/mL in the absence or presence of 20 μg/mL LysSPYS2. The control group and LysSPYS2-alone group are also shown. Data are presented as mean ± SEM (n = 3).

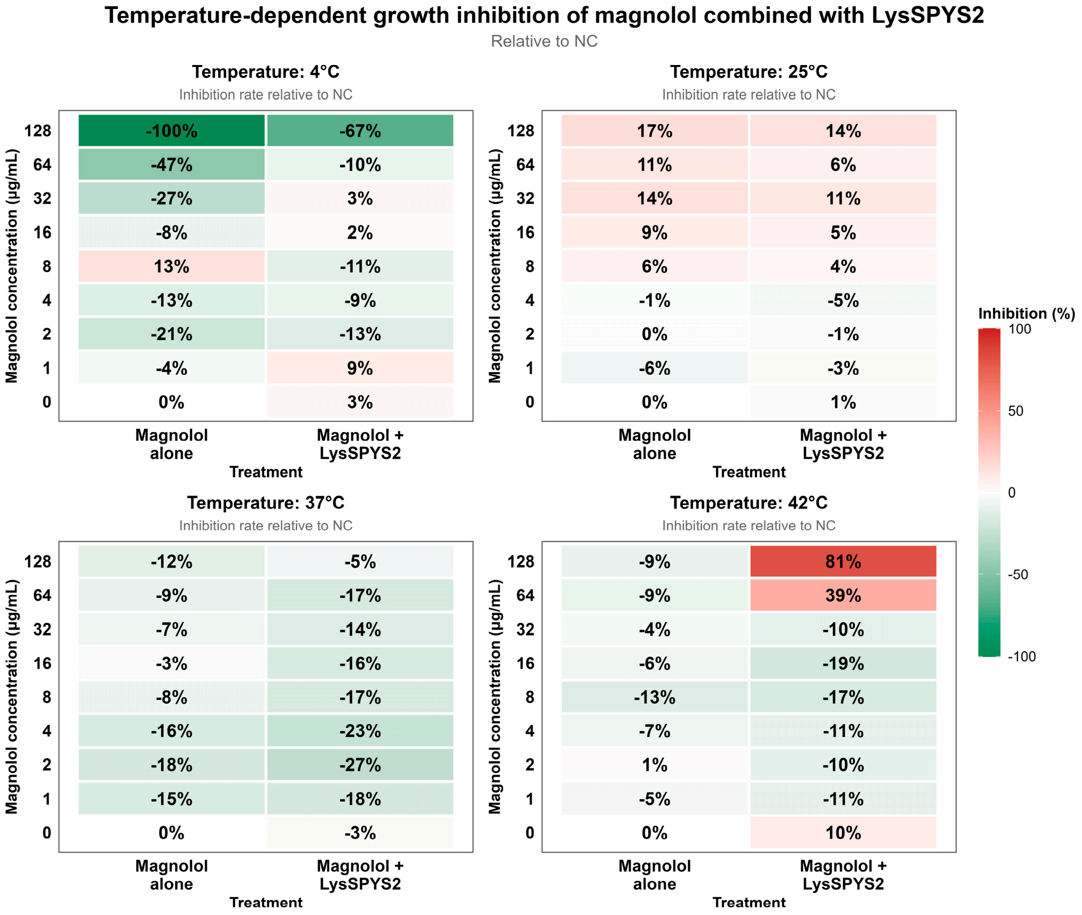

Figure S2. Temperature-dependent growth inhibition rates of magnolol combined with LysSPYS2 against *S. Typhimurium* ATCC 14028. Inhibition rates were calculated from OD_{600} values relative to the NC group at each temperature. In the "Magnolol alone" group, 0 μg/mL represents the NC group, while in the "Magnolol + LysSPYS2" group, 0 μg/mL represents LysSPYS2 treatment alone. Positive inhibition values indicate growth suppression, whereas negative values indicate increased bacterial growth relative to NC.
